# Rate of virological failure and HIV-1 drug resistance among HIV-infected adolescents in routine follow-up on health facilities in Cameroon

**DOI:** 10.1371/journal.pone.0276730

**Published:** 2022-10-26

**Authors:** Calixte Ida Penda, Magaly Moukoko Mbonjo, Joseph Fokam, Armando Blondel Djiyou Djeuda, Ngondi Grace, Francis Ateba Ndongo, Serge Bilong, Bertrand Eyoum Bille, Paul Koki Ndombo, Avelin Aghokeng, Alexis Ndjolo, Carole Else Eboumbou Moukoko

**Affiliations:** 1 Faculty of Medicine and Pharmaceutical Sciences, University of Douala, Douala, Cameroon; 2 Laquintinie Hospital of Douala, Douala, Cameroon; 3 Douala General Hospital, Douala, Cameroun; 4 Yaoundé Central Hospital, Yaoundé, Cameroon; 5 Faculty of Health Sciences, University of Buea, Buea, Cameroon; 6 Chantal BIYA International Reference Centre for Research on HIV/AIDS Prevention and Management (CIRCB), Yaoundé, Cameroon; 7 Laboratory of Parasitology, Mycology and Virology, Postgraduate Training Unit for Health Sciences, University of Douala, Douala, Cameroon; 8 Faculty of Medicine and Biomedical Sciences, University of Ngaoundéré, Ngaoundéré, Cameroon; 9 Mother & Child Centre (MCH), Chantal Biya Foundation, Yaoundé, Cameroon; 10 Faculty of Medicine and Biomedical Sciences, University of Yaoundé I, Yaoundé, Cameroon; 11 Central Technical Group, National AIDS Control Committee, Yaoundé, Cameroon; 12 MIVEGEC, Université de Montpellier, CNRS, IRD, Montpellier, France; 13 Centre Pasteur Cameroon, Yaoundé, Cameroun; Consejo Superior de Investigaciones Cientificas, SPAIN

## Abstract

The objective of this study was to determine the rates of virological failure (VF) and HIV drug resistance (HIVDR) amongst adolescents on antiretroviral Therapy (ART). A retrospectively designed study was conducted in 10 healthcare centers for adolescents living with HIV (ALHIV) in the two main cities of Cameroon (Yaoundé and Douala), from November 2018 to May 2019. Sociodemographic, clinical, therapeutic and laboratory parameters were collected from medical records. All enrolled ALHIV had viral load (VL) measurements following the national guidelines. All patients with a VL ≥ 1000 copies/ml were called to perform genotyping tests. The chi-square test was used to determine the factors associated with VF. Out of the 1316 medical records of ALHIV, we included 1083 ALHIV having a VL result. Among them, 276 (25.5%) were experiencing VF, and VF was significantly higher in ALHIV with suboptimal adherence (p<0.001), older adolescents (p<0.05), those who lived outside the city where they were receiving ART (p<0.006), severely immunocompromised (p<0.01) and started ART at infancy (p<0.02). Among the 45/276 (16.3%) participants with an available genotyping resistance testing (GRT) result, the overall rate of HIVDR was 93.3% (42/45). The most common mutations were K103N (n = 21/42, 52.3%) resulting in high-level resistance to Efavirenz and Nevirapine, followed by M184V (n = 20/42, 47.6%) and thymidine analog mutations (n = 15/42, 35.7%) associated with high-level resistance to Lamivudine and Zidovudine respectively. The high rate of VF and HIVDR among ALHIV regularly followed in health facilities in Cameroon highlights the need to develop interventions adapted to an adolescent-centered approach to preserve future ART options.

## Introduction

Infection by the human immunodeficiency virus (HIV) remains a major public health problem with 36.9 million people living with HIV (PLHIV) worldwide, of which about 1.8 million were adolescents aged 10 to 19 years old and nearly 90% of them resided in sub-Saharan Africa (SSA) in 2020 [1]. Intensified programs for the prevention of mother-to-child transmission of HIV (PMTCT) have led to a rapid decline of about 58% in new HIV infections among children (aged 0–14 years) since 2000. However, in adolescents aged 15 to 19, this reduction is slower [2]. The Joint United Nations Program on HIV/AIDS (UNAIDS) has set an ambitious target to reduce morbidity and mortality and increase the life expectancy of PLHIV, which stipulates that 95% of people living with HIV know their status, 95% of those who know their status are on antiretroviral therapy (ART) and 95% of those on ART have a suppressed viral load by 2025 [3]. It is therefore imperative to improve efforts amongst adolescents living with HIV (ALHIV) who are still lagging behind.

Adolescence is a period of physiological and psychological growth characterized by biological, sexual and identity development, as well as increasing social autonomy [4, 5]. Poor treatment adherence during adolescence has been reported for many chronic disease conditions, including HIV infection [6, 7]. Of note, ALHIV who start ART are less likely to adhere to treatment and remain in care [8]. Compared to adults, ALHIV may have a lot of challenges with ART when started at infancy or childhood, and are more at risk of treatment failure and HIV drug resistance (HIVDR) emergence. The emergence of HIVDR is a major challenge to the effectiveness of ART [9]. Accumulation of HIVDR is of greater concern among adolescents in SSA due to limited availability of viral load (VL) monitoring, late switch to second-line ART in case of treatment failure, and limited treatment options [10].

There is a scarcity of data on the virologic success and failure of ALHIV on ART [7], especially in central Africa. Most studies to date do not provide precise estimates of the rate of virological failure (VF) and success specific to adolescents [11]. Data on the frequency of the emergence of HIVDR on the effectiveness of available treatments are limited. Indeed, previous studies conducted in resource-limited settings have reported alarming rates of VF among HIV-infected adolescents, ranging from 10.4% in Myanmar to 29.0% in Thailand, 33.0% in Zimbabwe and 51.6% in Togo [12–15]. These high rates of VF were frequently associated with a high prevalence of drug resistance mutations (DRM), ranging from 67.0%, 90.4% and 94.4% in Zimbabwe, Tanzania and Togo respectively. These accumulations of DRM compromise the success of future treatment regimens and their survival [14–16].

In Cameroon, according to the National AIDS control program, approximately 48000 children and adolescents were living with HIV in 2017, among which 56.7% are receiving ART [17]. However, only 57.0% of children and adolescents receiving ART achieved viral suppression [18]. A previous study in this age group in a single region of Cameroon has reported low viral suppression rates among adolescents (53.3%) compared to children (75.8%) and adults (81.1%) [19].The aim of our work was to determine the rate of VF and HIVDR in HIV-infected adolescents on ART followed routinely on a larger scale in the two main cities of Cameroon.

## Materials and methods

### Study design and setting

We conducted a cross-sectional study from November 2018 to May 2019 using retrospective and prospective data collected in ten specialized treatment and care centers for ALHIV located equally in the two main cities of Cameroon (Yaoundé and Douala).

The study sites were selected based on the high number of HIV-infected adolescents on ART followed according to national recommendations. In Yaoundé, the five health facilities involved were the Centre mère & enfant de la fondation Chantal Biya (CME), Hôpital Général de Yaoundé (HGY), Centre Hospitalier Essos (CHE), Hôpital Gynéco-Obstétrique de Yaoundé (HGOPY) and Centre Hospitalier Universitaire de Yaoundé (CHU). While in Douala, the other 05 healthcare facilities selected were: Hôpital Laquintinie de Douala (HLD), Centre médical d’arrondissement de Soboum (CMA Soboum); Hôpital General de Douala (HGD), Hôpital de District Nylon (HD Nylon), Hôpital Saint Albert le Grand (HSALG).

### Study population and sample size

For the retrospective phase of the study, we extracted information from participants’ medical records and considered the following inclusion criteria: HIV-infected adolescents on ART for at least 6 months and followed up in one of the selected study sites. For the prospective phase, we obtained parental consent and adolescent assent according to national guidelines. To estimate the necessary sample size, we assumed a VF rate of 20% based on previous publications [20]. Therefore, we considered a minimum sample size of 246 adolescents as necessary to achieve a 95% confidence interval and a tolerable error of 5%.

### Study procedures and sample collection

A census of all medical records of HIV-infected adolescents on ART for at least 6 months was carried out in the 10 participating sites of the study. Socio-demographic characteristics (age, sex, place of residence), clinical (WHO stage, transmission route), therapeutic (previous and current ART regimens), and biological (VL and CD4 count if available) data were collected using a standardized questionnaire and/or after discussion with the adolescent or his guardian if necessary. Disclosure of HIV status was collected and categorized according to whether the disclosure was complete, partial, or not started. This was considered complete if the adolescent had gone through the full disclosure process and was aware of their HIV status, or partial if they were aware of having a chronic disease but the HIV was not named. Adherence assessment was based on keeping clinic appointments using medical records and collecting antiretroviral (ARV) drugs at the pharmacy on time using ARV dispensing records. For the estimation of VF, we considered the most recent VL value, and only VL tests performed within the last 12 months were included. A patient was considered in VF if his last VL value was ≥1000 copies/ml. Routinely, in case of unsuppressed viral load ≥1000 copies/ml (3.0 log10 copies/ml), adherence support was offered to patients and a change of ART was prescribed (when necessary), then VL monitoring was performed 3 months later according to national recommendations. Incomplete records of adolescents with no VL were excluded from the analysis.

### Viral load and drug resistance testing

VL testing was performed using the Abbott m2000RT platform (Abbott Laboratories, Des Plaines, IL, USA) according to the manufacturer’s instructions, with a detection limit of 40 copies/ml. All participants with a VL ≥1000 copies/mL were considered eligible for HIV drug resistance testing. They were all called to perform genotyping tests but a blood sample was collected only from those who accepted being part of this study and signed the informed consent/assent. A blood sample was collected in two 5 mL tubes containing ethylenediaminetetraacetic acid and the recovered plasma aliquots were stored at -80°C for subsequent confirmation of VL and genotyping resistance testing (GRT). Specimens of confirmed high plasma VL (≥1000 copies) were centralized at CIRCB, where drug resistance genotyping was performed using a previously published in-house protocol [21], which amplifies protease and of reverse transcriptase regions in the *pol* gene fragment (~1600 bp). The sequences were assembled and edited using Seqscape version 2.5 software and DRMs were interpreted using the Stanford HIV drug resistance algorithms database version 8.8 (http://www.hivdb.stanford.edu). HIV-1 subtyping was done using Bio-edit v.5.0.26 for sequence multiple alignment, COMET, REGA and BLAST for rapid subtyping, with confirmation of subtypes and recombinants by molecular phylogeny using MEGA IX.

### Statistical analysis

Sociodemographic, clinical and therapeutic data were collected using a pre-established questionnaire. The biological data studied were the frequency of VF and HIVDR in our study population. Indeed, VF was defined if a patient already had a VL ≥1000 copies/mL, while HIVDR was defined as the presence of a DRM to at least one ARV drug.

Data were entered and saved using CS Pro version 7 data entry software. The chi-square test was used to determine the association between VF and sociodemographic, clinical, laboratory and therapeutic characteristics. Statistical analysis was performed using SPSS v.23 and a p-value of less than 0.05 was considered statistically significant for the analysis.

### Ethical consideration

The study was approved by the Ethics Committee of the Faculty of Medicine and Biomedical Sciences of the University of Yaoundé I (N°165/UYI/FMSB/VDRC/CSD). Additional ethical clearances were obtained from The Regional Ethics Committee for Centre (CE N°1548-9/CRERSHC/2019) and Littoral (N°485-19/AAR/MINSANTE/DRSPL/BCASS) regions where the study was conducted. Administrative authorizations were also obtained from each of the health facilities involved in the study. Adolescents and their parents were informed of the purpose and process of the survey (background, goals, objectives, methodology, data confidentiality, and rights to withdraw from the study without prejudice), and signed informed consent was obtained from parents /guardians of adolescents in accordance with the Declaration of Helsinki and verbal assent of all adolescents in accordance with national guidelines prior to study inclusion. All patients were treated free of charge according to the treatment guidelines of the National HIV Control Program in Cameroon.

## Results

### Characteristics of the study population

**Fig 1:**
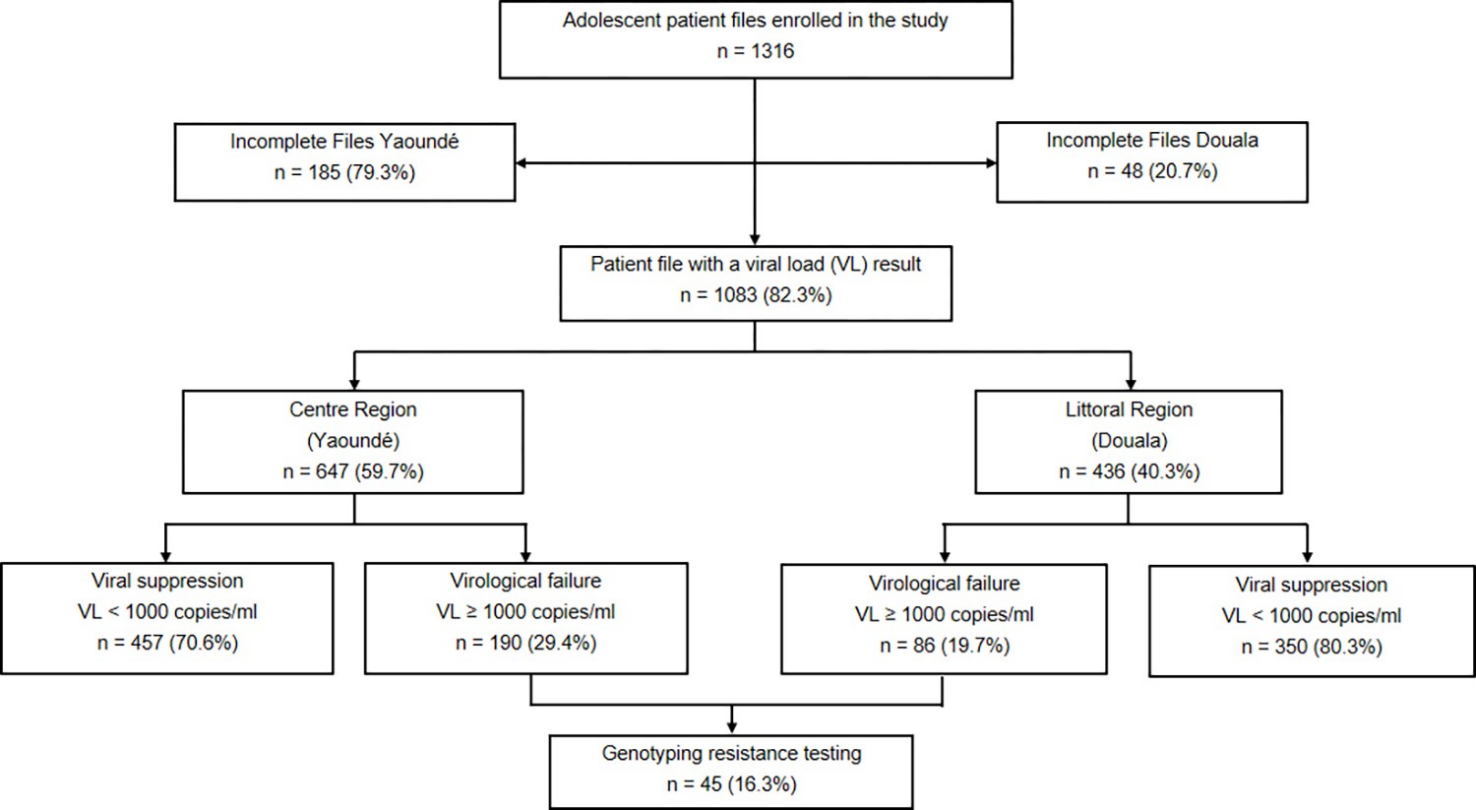
Flowchart of HIV-infected adolescents on ART followed in the Yaoundé and Douala study sites.

The participant inclusion flowchart is shown in Fig 1. Based on the inclusion criteria, we initially identified 1316 patients from medical records at the study sites, of which 1083 (82.3%) were included and 233 (17.7%) excluded for incomplete records with missing VL results. Participants were followed up in healthcare facilities located either in Yaoundé (n = 647/1083, 59.7%) or Douala (n = 436/1083, 40.3%) regions. Among the 1083 patients’ medical records included, 647 (59.7%) were from patients followed up in Yaoundé and 436 (40.3%) from Douala. Of them, a total of 276 adolescents (190 from Yaoundé and 86 from Douala) had a viral load ≥1000 copies/ml and genotyping resistance testing was performed for 45 of them (16.3%).

The median age of participants was 14 years [IQR: 12–17], and 591 (54.6%) were female. More than half of adolescents (n = 615/1083, 56.8%) were orphans of one or both parents. More than 8 in 10 adolescents (911/1083, 84.1%) partially (344; 26.1%) or completely (739; 56.2%) knew their HIV status. Of the 1083 participants, 1008 (93.1%) were infected by vertical transmission. The majority (899/1083, 83.0%) of the participants were receiving a first-line regimen and 89.5% had been on ART for more than 24 months; with a median time on ART of 7 years [IQR:2–11]. In our study population, 49.9% of adolescents had changed ART (either for treatment optimization, reduction of pill burden or switching to a second/third line regimen purposes), including 27.5% more than once. Unavailability of ART (62.5%) at the health center was the most common reason for switching, followed by discontinuity of follow-up (34.5%). At initiation, Nevirapine- or Efavirenz-based regimens were given to 998 (92.2%) participants. Up to 1044 adolescents (96.4%) respected their appointment and withdrawn their ARV drugs from the pharmacy on time. Table 1 shows the characteristics of our study population.

**Table 1:** Baseline characteristics of the study population and virological suppression levels.

Characteristics	Overall N = 1083		Virological suppression (VL < 1000 copies/ml) N = 807 (74.5%)		Virological failure (VL ≥ 1000copies/ml) N = 276 (25.5%)		*p*-value
	n	%	n	%	n	%	
City							
Yaoundé	647	59.7	457	56.6	190	68.8	< 0.001
Douala	436	40.3	350	43.4	86	31.2	
Category of healthcare facility ^a^							
Category 1- General hospitals	129	11.9	83	10.3	46	16.7	0.02
Category 2- Central hospitals	810	74.8	614	76.1	196	71.0	
Category 4- Districts hospitals	107	9.9	79	9.8	28	10.1	
Category 5- Arrondissement medical centers	37	3.4	31	3.8	6	2.2	
Age (years)							
10–14	563	52.0	435	53.9	132	47.8	0.05
15–19	520	48.0	372	46.1	144	52.2	
Gender							
Male	492	45.4	368	45.6	124	44.9	0.63
Female	591	54.6	439	54.4	152	55.1	
Parental life status							
Orphan	615	56.8	445	55.1	170	61.6	0.5
Non-orphan	468	43.2	363	44.9	105	39.4	
Caregiver relationship							
Parent	677	62.5	511	63.3	166	60.1	0.4
Non-parent	406	37.5	296	36.7	110	39.9	
Caregiver employment							
Employed	612	56.5	499	61.8	113	40.9	0.64
Unemployed	481	43.5	318	38.2	163	59.1	
Area of residence							
Out of town	179	16.5	119	14.8	60	21.7	0.006
In town	904	83.5	688	85.2	216	78.3	
Disclosure of HIV status							
Partial or complete disclosure	911	84.1	681	84.4	230	83.3	0.7
No disclosure	172	15.9	126	15.6	46	16.7	
Baseline CD4 count (cells/mm^3^) ^b^							
> 500	250	46.6	205	49.6	45	36.3	0.01
201–499	164	30.5	124	30.0	40	32.3	
< 200	123	22.9	84	20.4	39	31.4	
Past history of opportunistic infection							
Yes	243	22.4	175	21.7	68	24.6	0.34
No	840	77.6	632	78.3	208	75.4	
Current WHO stage							
Stage 1- asymptomatic infection	1059	97.8	794	98.4	265	96.0	0.04
Stage 2 or 3- symptomatic infection	24	2.2	13	1.6	11	4.0	
PMTCT exposure							
Yes	69	6.4	55	6.8	14	5.1	0.31
No	1014	93.6	752	93.2	262	94.9	
Age group on ART initiation (years)							
< 4	296	27.3	245	30.4	51	18.5	0.02
4–9	452	41.7	316	39.2	135	48.9	
10–14	281	25.9	198	24.5	83	30.1	
15–19	55	5.1	48	5.9	7	2.5	
Duration on ART (months)							
< 12	45	4.2	33	3.1	12	4.3	0.4
12–24	73	6.7	55	6.8	18	6.5	
> 24	965	89.1	719	89.1	246	89.1	
Adherence ^c^							
Yes	1044	96.4	804	99.6	240	87.0	< 0.001
No	39	3.6	3	0.4	36	13.0	
ART regimen at initiation ^d^							
AZT+3TC+NVP/EFV	390	36.0	291	36.1	99	35.9	
ABC+3TC+NVP/EFV	248	22.9	180	22.3	68	24.6	
TDF+3TC+EFV	251	23.2	181	22.4	70	25.4	0.12
ABC/AZT+3TC+LPV/r	84	7.8	67	8.3	18	6.5	
Other	109	10.1	88	10.9	21	7.6	

ART, antiretroviral treatment; PMTCT, prevention of mother-to-child transmission; VL, viral load; WHO, world health organization; AZT, Zidovudine; ABC, Abacavir; 3TC, Lamivudine; NVP, Nevirapine; EFV, Efavirenz; TDF, Tenofovir; LPV/r, Lopinavir boosted with ritonavir; D4T, Stavudine.

^a^ The healthcare facilities are organized into seven main categories in Cameroon. However, four (4) categories were represented in this study: category 1, general hospitals; category 2, central hospitals; category 4, districts hospitals and category 5, Arrondissement medical centers.

^b^ Data available for only n = 537 participants.

^c^ Evaluated by assessing the proportion of participants that respected their appointments and withdrew their drugs within time limits.

^d^ Other includes: D4T+3TC+NVP/EFV (n = 97) and TDF+3TC+NVP (n = 12).

### Virological outcomes and associated factors

Of the 1083 ALHIV, 807 (74.5%) had a suppressed viral load according to the WHO threshold (VL <1000 copies/mL), and 731/1083 (67.5%) had an undetectable viral load (VL <50 copies/mL). A total of 276 participants experienced VF, therefore representing a failure rate of 25.5% in our study population. The rate of VF varied according to the healthcare facility, ranging from 16% to more than 63% as shown in Table 2.

**Table 2:** Distribution of virological status according to the health facility.

Healthcare Facility	City	Complete files n (%)	Incomplete files n (%)	Viral suppression n (%)	Virological Failure n (%)
CHU	Yaounde	36 (64.3)	20 (35.7)	26 (72.2)	10 (27.8)
CHE	Yaounde	91 (56.1)	71 (43.9)	75 (82.4)	16 (17.6)
CME	Yaounde	450 (86.1)	73 (13.9)	313 (69.6)	137 (30.4)
HGY	Yaounde	19 (51.3)	18 (48.7)	7 (36.8)	12 (63.2)
HGOPY	Yaounde	51 (94.4)	3 (5.6)	36 (70.5)	15 (29.5)
HGD	Douala	23 (62.1)	14 (37.9)	14 (60.8)	9 (39.2)
HLD	Douala	269 (97.8)	6 (2.2)	226 (84.0)	43 (16.0)
CMA Soboum	Douala	37 (67.2)	18 (32.8)	31 (83.7)	6 (16.3)
HD Nylon	Douala	70 (93.3)	5 (6.7)	55 (78.6)	15 (21.4)
HSALG	Douala	37 (88.1)	5 (11.9)	24 (64.9)	13 (35.1)
Total		1083 (82.3)	233 (17.7)	807 (74.5)	276 (25.5)

CME, Centre mère et enfant de la fondation Chantal Biya; HGY, Hôpital Général de Yaounde; CHE, Centre Hospitalier Essos; HGOPY, Hôpital Gynéco-Obstétrique de Yaoundé; CHU, centre hospitalier universitaire de Yaounde; HLD, Hôpital Laquintinie de Douala; CMA Soboum, Centre médical d’arrondissement de Soboum; HGD, Hôpital Général de Douala, HD Nylon, Hôpital de District Nylon; HSALG, Hôpital Saint Albert le Grand.

Factors associated with VF included, being severely immuno-compromised at ART initiation with a CD4 count below 200 cells/mm3 (p = 0.01), having started ART between 4 and 9 years of age (p = 0.02), late adolescence (p = 0.05), followed up in a health facility located in Yaoundé (p < 0.001), followed up in a first category health facility according to the Cameroonian healthcare pyramid (p = 0.02), living out of the town in which they were receiving ART (p = 0.006), and late or absence drug withdrawal from the pharmacy (p < 0.001). However, no association was observed between VF and non-disclosure of HIV status, exposure to PMTCT, history of opportunistic infections, treatment regimens or duration on ART (Table 1).

### HIV drug resistance mutations

A total of 45/276 (18.1%) plasma samples successfully underwent genotyping resistance testing (GRT). Of the 45 sequences, 42 (93.3%) harbored resistance to at least one ARV drug, with nearly 90% (40/45) having intermediate or high-level HIV drug resistance. About 71.1% of adolescents who underwent GRT had multiclass ARV resistance mutations. Moreover, 60% (27/45) carried a resistant virus to both NRTIs and NNRTIs and 11% (5/45) carried at least one drug resistance mutation (DRM) to three classes, namely NRTI, NNRTI and PI. Two participants (4.4%) had resistance only to NRTIs, seven (15.6%) harbored resistance only to NNRTIs and one (2.2%) was resistant only to PIs. Resistance to all drugs in the same class for NNRTIs and NRTIs was observed in 8/45 (17.8%) and 2 (4.4%) HIV-infected adolescents, respectively (Table 3).

**Table 3:** HIV drug resistance profile among adolescents failing treatment (VL ≥ 1000 copies/ml).

Variables	n	%
Total specimens eligible for GRT	276	
GRT successfully performed	45	16.3
Virus resistance level		
Susceptible	3	6.7
Low level	2	4.4
Intermediate level	29	64.4
High-level	11	24.5
Frequency of HIV drug resistance by class		
NRTI	2	4.4
NNRTI	7	15.6
PI	1	2.2
NRTI + NNRTI	27	60.0
NRTI + NNRTI + PI	5	11.1
None	3	6.7
Resistance to all drugs in one class		
NRTI	2	4.4
NNRTI	8	17.8
None	35	77.8

GRT, genotyping resistance testing; NNRTI, non-nucleoside reverse transcriptase inhibitor; NRTI, nucleoside reverse transcriptase inhibitor; PI, protease inhibitor

**Fig 2:**
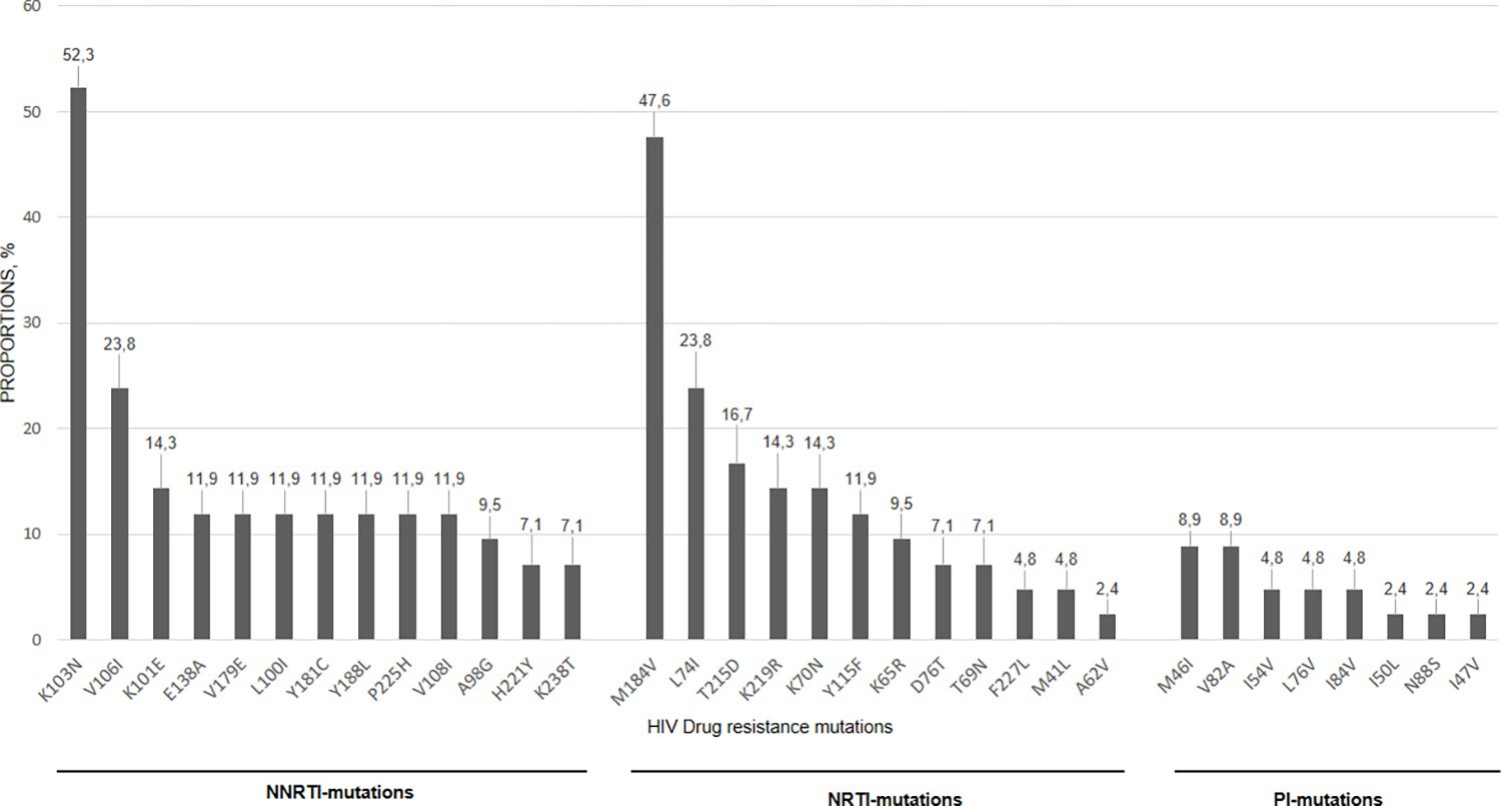
Proportion (%) of the different drug-resistant mutations. Note. Proportions of HIV drug-resistance mutations identified among adolescents in virological failure. Drug resistance mutations are presented by decreasing order of expression level. The figure shows the different mutations according to different classes of drugs: nucleoside reverse transcriptase inhibitor (NRTI), non-nucleoside reverse transcriptase inhibitor (NNRTI) and protease inhibitor (PI).

Drug-resistance mutations (DRMs) were found in the three classes of drugs assessed. The DRMs frequently found were K103N leading to high-level resistance to Efavirenz (EFV) and Nevirapine (NVP) in 22 of 42 adolescents (52.3%), and M184V, triggering high resistance to Lamivudine (3TC) and Emtricitabine (FTC) in 20 of 42 (47.6%) ALHIV. Other NNRTI DRMs were V106I (n = 10/42, 23.8%), K101E (n = 6/42, 14.3%), and Y181C, Y188L, E138A, V179E, L100I, P225H, V108I (n = 5/42, 11.9%), having low to high resistance against EFV and NVP alone or in combination. Additionally, thymidine analog mutations (TAM) resistance, including T215D, K219R, and M41L (n = 15/42, 35.7%), and non-TAMs such as L74I (n = 10/42, 23.8%) and K70N (n = 6/42, 14.3%) were other DRMs associated with additional reductions in susceptibility to NRTIs, particularly Abacavir (ABC), Zidovudine (AZT) and Stavudine (D4T). The two main DRMs for PI were M46I and V82A found in 4 of 42 adolescents (9.5%), and associated with reduced susceptibility to both Atazanavir (ATV) and Lopinavir (LPV). The proportion of each DRM is shown in Fig 2.

**Fig 3:**
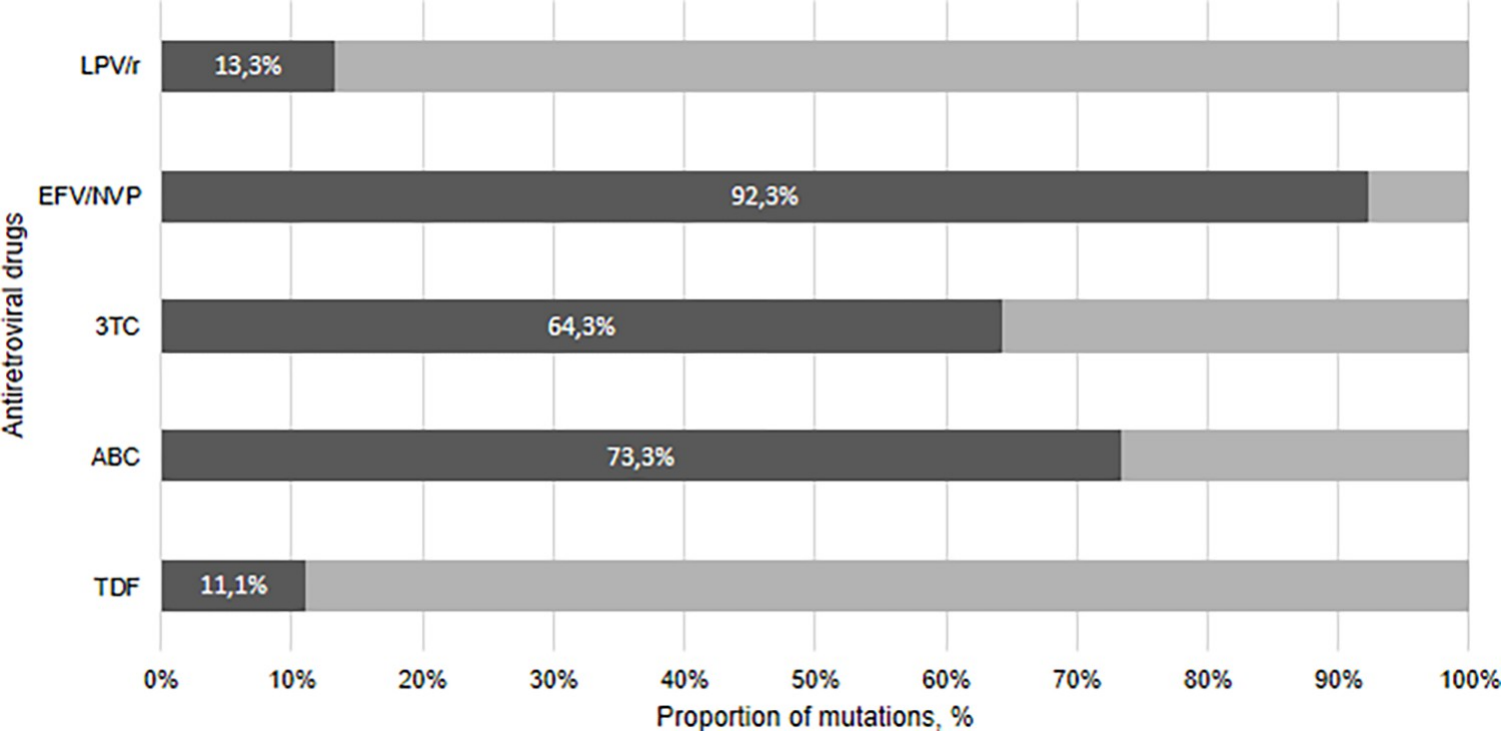
Resistance level according to the major antiretroviral drugs currently used by the participants who underwent genotyping testing (n = 42 participants). Note. Resistance levels are presented by increasing proportion of mutations (%) conferring resistance to the drugs currently used by the study participants. Major antiretroviral drugs are indicated: TDF, Tenofovir; LPV/r, Lopinavir boosted with ritonavir; 3TC, Lamivudine; ABC, Abacavir; EFV, Efavirenz; NVP, Nevirapine.

Of the 42 HIV-infected adolescents carrying DRMs to main ARVs used, we observed that 92.3% (n = 24/26) of the participants in our study who had received therapeutic regimens based on EFV or NVP were very resistant to these ARVs. Similarly, 73.3% (n = 11/15) of adolescents who received ABC had high-level resistance to Abacavir while 64.3% (27/42) were resistant to 3TC, 13.3% (2/15) at LPV/r and 11.1 (n = 3/27) at TDF (Fig 3).

A total of 8 viral clades were found, among which 4 recombinant forms and pure subtypes. Of note, CRF02_AG was the most prevalent clade (55.6% [25]), followed by 13.3% (6) subtype A, 11.1% (5) subtype F2, 9% (4) subtype G, 4.4% CRF13.cpx, and others at 2.2% (1) each (subtype D, CRF11.cpx, and CRF18.cpx). Moreover, the genetic diversity had no major effect on drug resistance profile, when comparing the rate of HIVDR among CRF02_AG (92% [23/25]) versus non-AG sequences (95% [19/20]), p = 1.000.

## Discussion

To our knowledge, this is one of the few studies evaluating VF and HIVDR in a large cohort involving more than a thousand HIV-infected adolescents in Central Africa and precisely in Cameroon. Our results showed a high rate of VF (25.5%), representing a quarter of ALHIV in our study population. This strong trend has been reported in previous studies conducted in similar settings. Makadzange et al., in Zimbabwe in 2012 in a study involving 418 adolescents reported a 33% rate of virologic failure among ALHIV on ART [14]. Similarly, alarming rates of VF have been reported among ALHIV, ranging from 34.2% (n = 92/270) in Cameroon in 2019, 41.8% (n = 69/165) in Côte d’Ivoire in 2017 to 51.6% (n = 86/167) in Togo in 2014 [15, 21, 22]. However, the distribution of VF was very heterogeneous, and varied from one health facility to another. Surprisingly, in contrast to a study conducted by Jobanputra et al. in Swaziland who showed that the management of pediatric HIV in primary care health centers was a risk factor for VF [23], VL suppression was significantly low for ALHIV followed in the first level category health facilities of the healthcare pyramid where specialist physicians were more present. This result might be explained by the weak application of task shifting at the tertiary level compared to those followed at the lower levels.

VF was higher among ALHIV who were poorly adherent to ART, were living outside the city where they received ART, were severely immunocompromised (<200 cells/mm^3^) at ART initiation and had started ART early in childhood (between 4–9 years old). A similar trend was observed in a previous study in Zimbabwe where a decreased risk of VF failure was associated with progressive increases in age at ART initiation [14]. This suggests a certain weariness regularly encountered in ALHIV on ART from early childhood, increased by the problems of late disclosure of HIV status. ART observance among ALHIV should be continuously boosted by therapeutic educators throughout the ARV intake. In addition, the increased risk of VF in ALHIV living out of the town where they received ART could be due to the long distance traveled to reach the health facility and the high cost of transport which constitutes an obstacle to access to health services. Decentralization of HIV care for children and adolescents must continue in our context to facilitate the provision of differentiated services in health centers close to communities in accordance with WHO guidelines [24].

The median age of ART duration in our study was 7 years, suggesting significant exposure to first-line regimens based on reverse transcriptase inhibitor with a low genetic barrier. Sub-optimal adherence to ART is often associated with VF in children and adolescents on ART [22, 25, 26].

Maintaining good adherence among ALHIV depends on the interplay of medication, psychosocial, health system and health worker factors [16]. Biologically, poor adherence to ART is manifested by low plasma concentrations of antiretroviral insufficient to produce the expected therapeutic effect, thus favoring the development of DRMs [25, 27]. We observed a high prevalence (n = 42/45, 93.3%) of HIVDR in ALHIV with treatment failure. Alarming rates of HIVDR have already been reported in similar settings, particularly in Tanzania and Togo, where HIVDR was found in 90.4% and 94.4% of ALHIV with VF respectively [14, 15]. More recently, a study in Cameroon also showed high levels of HIVDR, exceeding 90% in both urban and rural settings [21]. The high proportion of multiclass ARV drug resistance observed in more than 70% of treatment failure patients was similar to that reported in Zimbabwe (79%) and remains of concern for future treatment options [28]. A high proportion of DRM to NNRTIs has been observed in ALHIV due to long exposure to first-line treatment based on NRTIs and NNRTIs and their low genetic barrier [29], the same phenomenon has been reported in a study national in adults in Cameroon [30]. Substitution of NNRTIs with the newly recommended integrase strand transfer inhibitors (INSTIs) Dolutegravir (DTG)-based regimen with a high resistance barrier may have a definite impact on NRTI resistance [31, 32]. Although emerging data report the efficacy of DTG-based regimens in patients with NRTI resistance, real data is needed, especially in ALHIV [33, 34].

The most common DRMs included the NNRTI K103N mutation (51.1%) and the NRTI M184V/I mutation (46.7%), followed by thymidine analog resistance mutations (TAM, 35.7%). This mutation profile is correlated with the known mutations induced by the drugs used in our context. Indeed, these results were consistent with previous reports showing high levels of ART resistance and an accumulation of resistance mutations in HIV-infected children and adolescents receiving Lamivudine, Nevirapine, or Efavirenz as the backbone of first-line ART regimens [10, 14–16, 35]. We found a few major DRMs including V82A and M46I with a percentage of 8.9% and a low percentage of ALHIV having a triple DRM class (11.1%) comparable to a study conducted in Madrid which found 9.1% Triple Class DRM [36].

The interest of this study lies in the inclusion of a large number of HIV-infected adolescents followed up in the two main cities in Cameroon. The results allow us to have a trend of routine monitoring of ALHIV on ART in the two largest cities of Cameroon and can be used by the program to improve the quality of care. Nevertheless, the main limitation of this study is related to the fact that our study was carried out in urban areas where access to health services and biological monitoring is greater than in the rest of the country. A national study would be necessary to better estimate the effectiveness of ART and the evaluation of resistance to HIV. On the other hand, we may have underestimated the VF because of the retrospective nature of this study by excluding the records of patients without VL. Finally, the low rate of successful genotyping has limited our understanding of mutation patterns in this age group.

## Conclusion

The rate of VF and HIVDR was high among ALHIV routinely followed in health centers in Cameroon. Strategies based on an approach centered on ALHIV and better therapeutic education could reduce the emergence of resistance within this vulnerable population.

## Abbreviations

PLHIV: people living with HIV
HIV: human immunodeficiency virus
UNAIDS: United Nations Program on HIV/AIDS
ART: antiretroviral therapy
PMTCT: prevention of mother-to-child transmission of HIV
SSA: sub-Saharan Africa
ALHIV: adolescents living with HIV
HIVDR: HIV drug resistance
VL: viral load
VF: virological failure
DRM: drug resistance mutations
CME: Centre mère & enfant de la fondation Chantal Biya
HGY: Hôpital Général de Yaoundé
CHE: Centre Hospitalier Essos
HGOPY: Hôpital Gynéco-Obstétrique de Yaoundé
CHU: Centre Hospitalier Universitaire de Yaoundé
HLD: Hôpital Laquintinie de Douala
CMA Soboum: Centre médical d’arrondissement de Soboum
HGD: Hôpital General de Douala, HD Nylon, Hôpital de District Nylon
HSALG: Hôpital Saint Albert le Grand
GRT: genotyping resistance testing
NNRTI: non-nucleoside reverse transcriptase inhibitor
NRTI: nucleoside reverse transcriptase inhibitor
PI: protease inhibitor
TAM: thymidine analog mutations
INSTIs: integrase strand transfer inhibitors
